# Effects of switching ticagrelor to clopidogrel on cardiovascular outcomes in patients with acute coronary syndrome: Erratum

**DOI:** 10.1097/MD.0000000000013949

**Published:** 2018-12-28

In the article, “Effects of switching ticagrelor to clopidogrel on cardiovascular outcomes in patients with acute coronary syndrome”,^[1]^ which appeared in Volume 97, Issue 48 of *Medicine*, Dr. Jianping Liu's name appeared incorrectly as Lin Liu.
